# Inkjet printing technology for increasing the I/O density of 3D TSV interposers

**DOI:** 10.1038/micronano.2017.2

**Published:** 2017-04-10

**Authors:** Behnam Khorramdel, Jessica Liljeholm, Mika-Matti Laurila, Toni Lammi, Gustaf Mårtensson, Thorbjörn Ebefors, Frank Niklaus, Matti Mäntysalo

**Affiliations:** 1 Department of Electronics and Communications Engineering, Tampere University of Technology, Korkeakoulunkatu 3, Tampere 33720, Finland; 2 Silex Microsystems AB, Bruttovägen 1, Järfälla/Stockholm SE-175 26, Sweden; 3 Micro and Nanosystem, KTH Royal Institute of Technology, Osquldas väg 10, 10044 Stockholm, Sweden; 4 EMSL, Chalmers University of Technology, Kemivägen 9, 41296 Göteborg, Sweden; 5 Mycronic AB, Nytorpsvägen 9, 18303 Täby, Sweden

**Keywords:** heterogeneous three-dimensional (3D) integration, inkjet printing, interposer, microelectromechanical system (MEMS), reliability, super inkjet (SIJ), through silicon via (TSV)

## Abstract

Interposers with through-silicon vias (TSVs) play a key role in the three-dimensional integration and packaging of integrated circuits and microelectromechanical systems. In the current practice of fabricating interposers, solder balls are placed next to the vias; however, this approach requires a large foot print for the input/output (I/O) connections. Therefore, in this study, we investigate the possibility of placing the solder balls directly on top of the vias, thereby enabling a smaller pitch between the solder balls and an increased density of the I/O connections. To reach this goal, inkjet printing (that is, piezo and super inkjet) was used to successfully fill and planarize hollow metal TSVs with a dielectric polymer. The under bump metallization (UBM) pads were also successfully printed with inkjet technology on top of the polymer-filled vias, using either Ag or Au inks. The reliability of the TSV interposers was investigated by a temperature cycling stress test (−40 °C to +125 °C). The stress test showed no impact on DC resistance of the TSVs; however, shrinkage and delamination of the polymer was observed, along with some micro-cracks in the UBM pads. For proof of concept, SnAgCu-based solder balls were jetted on the UBM pads.

## Introduction

**Figure 1:**
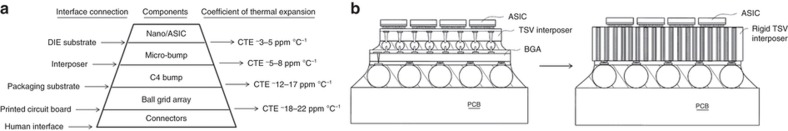
(**a**) Interface pyramid showing the components in a conventional package, along with the estimated CTEs of the involved substrates. (**b**) Introducing a rigid 3D TSV interposer (right) to bridge the mismatch of the CTE values of the PCB board and the Si application-specific integrated circuit (ASIC) dies^3^. CTE, coefficients of thermal expansion; PCB, printed circuit boards; 3D TSV, three dimensional through silicon via.

One of the major technology drivers in microelectromechanical systems (MEMS) is to incorporate increasing numbers of functionalities within the same foot print. This increase in functionalities directs the focus toward miniaturization, multichip module packages, and higher levels of integration of integrated circuits (ICs) and MEMS^1,2^. The use of different materials, such as silicon (Si), for MEMS and ICs chips and polymer laminates for printed circuit boards (PCB) in a multichip module, results in major challenges caused by the mismatch of the coefficients of thermal expansion (CTE) of the involved materials, different I/O densities between the components, and resulting stresses, as depicted in Figure 1a^3^. To overcome these problems, silicon interposer substrates with through-silicon vias (TSVs) can be introduced as an interface between the PCB and the IC and MEMS dies, as illustrated in Figure 1b^4^. An interposer with TSVs provides electrical connections through the interposer substrate and enables vertical stacking of the chips, shorter interconnect lengths, reduced size of the module, as well as improved energy efficiency of the module^2,5^. The interposer provides a possibility to integrate dies that are manufactured with different technologies, such as memory, power, logic, MEMS, and radio frequency (RF). The use of interposer substrates with TSVs is often referred to as 2.5D integration^2,4^.

TSVs with electrically conductive Si via cores have been realized by several MEMS manufacturers and foundries^6^. For these Si TSVs, trenches are etched in a heavily doped Si substrate, insulated with SiO_2_, and then filled with non-conductive poly-Si. By using TSVs with electrically conducting cores made of single-crystalline Si, a perfect match of the CTE values of the via cores and the Si substrate is obtained, thereby effectively eliminating potential reliability issues. However, Si-based TSVs have resistances in the range of 0.5–1 Ω and thus can only be used for low-frequency applications because the high resistance in the TSVs causes significant resistance-capacitance (RC) delay and signal decay.

**Figure 2:**
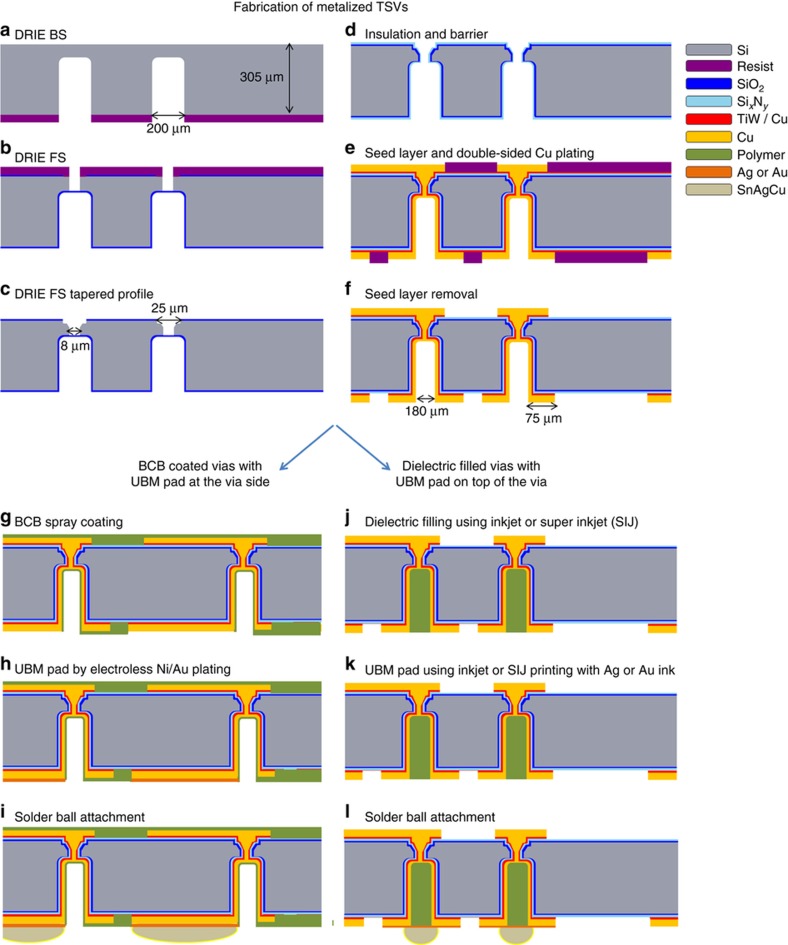
(**a**–**f**) Process flow for the TSV fabrication^9^. (**g**–**i**) Conventional fabrication approach with solder balls placed next to the via holes, resulting in low I/O densities, as in Ref. 10. (**j**–**l**) Approach demonstrated in this work with TSVs filled with inkjet-printed dielectric polymer and solder balls placed in the same area as the TSVs to increase the I/O density. I/O, input/output; TSV, through silicon via.

Thus, to obtain high electrical conductivity (low resistance) and thereby low losses for RF applications or other applications, TSVs with conductive metal cores, for example, made of Cu or Au, are needed^2^. The drawbacks of incorporating such metals are the reliability issues due to the different CTE values of the involved materials, for example, Cu or Au and Si. The difference in the CTE values of Cu (16.7 p.p.m. °C^−1^) and Si (2.5 p.p.m. °C^−1^)^5^ will induce mechanical stress at the material interfaces^7^. If vias are completely filled with Cu, then cracks in the Si can emerge during heating^8^. To avoid the formation of cracks in the Si substrate, the inner walls of the TSV holes can be conformally coated by conventional Cu plating, thereby creating hollow metal TSVs (Figures 2a–f)^9^. Thus, the empty space at the center of the vias allows the Cu to expand when heated, thereby minimizing the risk of crack formation in the Si substrate. In addition, a conformal Cu film limits the time consuming and expensive Cu plating, and reduces material consumption. Such a TSV fabrication process (Figures 2a–i) was for example reported in Refs. 9 and 10, where the Cu-metalized TSVs are finally coated with a thin layer of benzocyclobutene (BCB) to achieve passivation and to realize a masking layer for the under bump metallization (UBM) layer. The UBM pads were placed next to the TSVs, followed by solder ball attachment, creating a solder ball pitch of 1 mm. By this method, it is possible to fabricate one I/O per mm^2^ with proven reliability when exposed to temperature cycling (−40 °C to 125 °C) of more than 1000 cycles^8^. However, the drawback of creating hollow TSVs is that they require a comparably large foot print, resulting in low I/O densities. There are two potential approaches to tackle the increased foot print: the diameter of the TSVs can be reduced and/or the UBM pads and the solder balls can be placed directly on top of the TSVs. By decreasing the TSV diameter, the aspect ratio of the vias will be increased, and practical limitations for conformal metal seed layer deposition inside the high-aspect ratio via holes by physical vapor deposition (PVD) or chemical vapor deposition (CVD) technologies are quickly reached. More complex and expensive deposition techniques, such as metalorganic-CVD or atomic layer deposition (ALD), are then required^11^. An alternative metallization method may be to use additive technologies, such as metal inkjet printing, to fill the high-aspect ratio TSVs. This technology requires fewer process steps as well as less waste material compared to conventional processes, such as electroplating. Inkjet printing of planar structures, such as conductive traces and large area devices (for example, antennas, electrodes, and photovoltaics), has successfully been demonstrated. However, in the case of partial metallization of the vias, metal inkjet printing technology is not sufficiently mature (especially with Cu ink) for volume production and therefore not a viable solution for via metallization^12–18^. An alternate approach is to take advantage of the empty space in the Cu-plated hollow TSVs and place the UBM pads and the solder bumps on top of the TSVs, as shown in Figures 2j–l, instead of placing the bumps next to the TSVs, as described in Ref. 10. For this purpose, the hollow vias must be filled with a suitable dielectric material with a low CTE to match the CTE of the Si. Previously, polymer filling of TSVs using spin coating of liquid polymers and vacuum-assisted lamination of dry film resists have been investigated in Refs. 19–21. The drawback with using spin-coating or vacuum lamination of polymers is the extensive optimization required to remove air bubbles that can become trapped in the polymer, potentially causing reliability problems. In addition, the topography of wafers with hollow TSVs strongly depends on the via density, thereby further complicating process optimization of the coating processes. Using inkjet printing, each via is filled separately, thereby eliminating the risk of non-uniform coatings over the wafer. Furthermore, targeted drop-by-drop filling with inkjet technology in combination with vacuum drying reduces the risk of void formation.

Therefore, in this work, we demonstrate the filling of hollow metal TSVs for interposer applications by two different printing methods, that is, inkjet and super inkjet (SIJ), using an inkjettable UV-curable hybrid polymer. We study cross sections of the resulting TSVs and electrically tested the filled TSVs before and after temperature cycling stress testing. Furthermore, we study the feasibility of printing metal pads, consisting of either Ag or Au, on top of the dielectric layer as the UBM layer. In addition, solder balls were dispensed on the UBM pads as a proof of concept. The proposed approach, compared to the technology used in Ref. 10, enables four times higher I/O densities without using the next-generation TSV node. This has significant advantages because moving to the next-generation TSV node would involve smaller TSV dimensions with significantly increased fabrication challenges.

## Materials and methods

### Fabrication of metalized TSVs

The fabrication of the metalized TSVs was executed on 200 mm diameter and 305-μm-thick Si wafers (3–10 kΩ cm). The use of rigid Si interposers that are 300–430 μm thick avoids the challenges involved in handling ultrathin wafers, including the steps of bonding and debonding of the interposer substrate to temporary carrier wafers^2^. In addition, a thicker interposer substrate will better accommodate the stresses and reduce the stress gradient across the substrate, thereby reducing the wafer bow^3^. The via holes are etched by deep reaction ion etching on both the frontside (FS) and the backside (BS) of the wafer, as shown in Figures 2a and b. To be able to close the via holes and create hermetically sealed TSVs by plating, the FS vias are etched with tapered structure, as shown in Figure 2c. The FS vias are 25±10 μm deep, the surface openings are 25±5 μm in diameter, and the bottom openings of the vias on the FS are 8±3 μm in diameter. SiO_2_ is used as an insulation layer, Si_*x*_N_*y*_ as a wafer bow stress compensation layer, and a TiW/Cu layer is used as a barrier and seed layer, as shown in Figure 2d^5^. A Cu-plating process was used to fill the FS vias and, in the same step, conformally plate a layer of Cu on the inner walls of the BS via holes. Figure 2e shows the Cu redistribution layer (RDL) on both the FS and BS of the wafer, which was plated in the same Cu-plating step. This combined Cu deposition process for both the via fill and the RDL eliminates metal interfaces between the vias and the RDL, which otherwise can cause poor electrical contacts and conductive losses, especially in RF applications. In the final step, the resist and the Cu/TiW seed layer are removed, as illustrated in Figure 2f^9^.

### Material selection for TSV filling

To select a suitable dielectric material for filling the TSVs, four critical key material parameters must be considered for the target application: (1) Low-k to obtain good RF properties, even at frequencies in the GHz range, (2) low Young’s modulus to minimize the introduced stress levels, (3) CTE of the dielectric that is as close as possible to the CTE of Si and Cu to minimize the induced stress levels in the TSV interposer during the soldering process and during temperature cycling, and (4) low material shrinkage during the curing process. Considering all of these factors, we selected InkOrmo (micro resist technology GmbH, Berlin, Germany), which is an inkjettable UV-curable hybrid polymer with a low Young’s modulus of ~1 GPa, low-volume shrinkage and high thermal stability of up to 300 °C (short term) and 270 °C (long term), which is higher than the typical bonding temperature (217 °C) for the Sn_96.5%_Ag_3%_Cu_0.5%_ alloy paste. In addition, the heat resistance of the InkOrmo polymer is higher than typical peak temperatures (250 °C) of reflow processes used with the SnAgCu alloys. The low Young’s modulus of the polymer (~100 times less than Cu-plated coverage) results in substantially reduced thermal stresses in the interposer compared to the thermal stresses induced by typical vias that are completely filled with a metal, despite that the CTE of the InkOrmo polymer is not perfectly matched to the substrate material. Table 1 presents the detailed specifications of the InkOrmo material.

### TSV filling and UBM deposition with inkjet

A Dimatix DMP-2800 inkjet printer (Fujifilm, Lebanon, New Hampshire, USA) equipped with a nominal 10 pL cartridge (DMC-11610) was used for printing the precursor of the polymer dielectric into the via holes. The actual inner diameter of the BS via holes is 180 μm after the 10 μm Cu liner deposition, and the depth of 270 μm. For the printing and filling trials, a square pattern including 11×11 droplets per pixel (121 in total) with a resolution of 5080 dpi (drop space of 5 μm) was prepared.

The interposer structure was flushed with isopropanol and heated to 200 °C for 5 min and then cooled to room temperature before drop deposition as recommended by the ink supplier. First, a total of 20 layers were printed in two rounds with drying of the ink (evaporation of solvent) in a vacuum oven after each round. The printing parameters are listed in Supplementary Table S1. During the filling process, the printing parameters were stable, and the jetting direction of the droplets was straight over time. Initial experiments showed that the printed material volume was not sufficient for complete filling of the hollow TSVs. Thus, instead of two printing rounds, 26 layers were printed in three rounds (10+10+6) with two phases of drying in a vacuum oven (30 min at 65 °C) after the first and second rounds of printing. The 26 layers are equal to ~3000 droplets, which can be compared to the theoretically calculated 700 droplets comprising the polymer precursor and solvent required to fill the vias. This difference shows the effect of evaporation of the solvent from the polymer precursor and shrinkage of the polymer because of baking and curing of the material in the subsequent filling rounds. The lowest available pressure (10^−2^ mbar) was used in the vacuum oven to avoid the formation of pockets with trapped air inside the via holes during the drying steps (solvent evaporation). After the final drying step, the dielectric polymer in the via holes was UV-cured for ~20 s before heating the sample to 150 °C for 3 h on a hotplate to hardbake the polymer. During printing of the polymer into a single via hole, there was no delay between printing the first layers; however, during printing of the last layers, a delay was required for solvent evaporation. Because printing one layer by filling the whole TSV interposer with tens of vias takes 10 min and 40 s, no additional delay was required for solvent evaporation.

After filling the vias with the dielectric, a conventional inkjet was used to print Ag UBM pads on top of the vias using an established and reliable Ag ink, NPS-J silver ink from Harima Chemicals, Tokyo, Japan (particle size: 12 nm, metal content: 65 wt% and solvent: Tetradecane), as a proof of concept. The pads were circular with a diameter of 300 μm, overlapping 75 μm with the Cu collar of the TSV for connection. Ni-based ink may be a better choice because there is a risk for dissolving Ag pads into the solder balls during solder ball formation and ball bonding. However, at the time, no suitable inkjettable Ni ink was available for this experiment. Copper ink could be another alternative option; however, in our experience, Cu annealing processes are relatively complex, typically requiring photonic or thermal annealing in inert atmosphere with forming gas^22,23^.

### TSV filling and UBM deposition with SIJ

As a potential alternative, SIJ^24^ (SIJ-S050) manufactured by SIJTechnology, Inc. (Tsukuba, Japan) was also investigated for dispensing the dielectric material inside the via holes using a needle-shaped nozzle. SIJ is a commercial electrohydrodynamic inkjet printing technology that allows for sub-femtoliter droplet deposition with high precision. In our SIJ set-up, the nozzle is placed over a via hole to dispense the dielectric material and completely fill the hole with the dielectric before the nozzle is moved to the next hole. Because of the shrinkage of the dielectric polymer during the prebake in the vacuum oven, filling is performed in several rounds until the hollow TSV is completely filled. After finishing the filling process, the final prebake (60–90 °C for 15–30 min), UV curing (500–1500 mJ cm^−2^), and hard baking (150 °C for 3 h) was executed. Because Ag UBM pads printed by inkjet printing showed non-uniform coverage on top of the TSVs, SIJ was used to dispense Au ink during a spiral movement of the nozzle on top of the TSVs, starting at the edge of the copper collar and moving to the center. The detailed printing parameters are listed in Supplementary Table S2. The UBM pads were printed with Au ink (UTDots Inc., Champaign, Illinois, USA) and subsequently sintered in an oven at 250 °C for 1 h.

### Reliability testing by temperature cycling

Temperature cycling reliability testing was performed after the dielectric filling of the metal TSVs to benchmark against the initial reliability tests performed on the standard hollow metal TSVs^10^. The TSVs used for our experiments are identical (taken from the same wafer batch) to the TSVs used in Ref. 9. The resistances of these TSVs were measured to be on the order of 10.3 mΩ per via before filling the hollow vias with the dielectric polymer and placing the metal on top of the filling. The insulator does not affect the resistance, and the metal coverage thickness is two orders of magnitude smaller than the via structure. Thus, by its very nature, these additions have a minimal effect on the via resistance.

**Figure 3:**
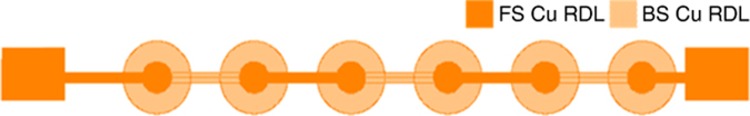
Via daisy chain structure. Two probes land on the probing pads on the left and the right side of the via chain.

The resistance of a via daisy chain was measured before and after the temperature cycling stress test (see Table 2) after 250, 500, 750, and 1000 cycles using a Keithley 2400 sourcemeter attached to a probe station (two-probe DC measurement). The overall measured resistance was ~2 Ω, which includes six vias in the chain, Cu RDLs connecting the vias and measurement pads, contact resistances, and cable resistances of the measurement set-up. The cable had the highest contribution to the overall resistance. However, the main purpose with the two-probe measurement was to examine the via chains for possible open circuits caused by damage of the plated copper after the temperature cycling reliability test. Figure 3 illustrates the daisy chain with pads on the left and right side designated for landing the probes to perform the electrical measurements.

After the electrical measurements, one via from each sample (three vias in total) was selected for preparing via cross sections using broad ion beam (BIB) technology to introduce the least possible damage to the samples. The cross sections were milled using 6 kV argon ions and an ~100-μm-thick layer was removed (half of the via). Next, scanning electron microscope (SEM) was used to characterize the cross sections to determine how the temperature cycling affects the dielectric filling inside the TSVs.

For the SIJ-printed Au UBM pads on top of the vias, the same reliability test was performed. The sample was imaged by SEM after 250 and 500 temperature cycles to evaluate the effect of temperature cycling on the pads.

### Solder ball attachment

The solder ball attachment on the Ag UBM pads was performed using a novel jetting technology (MY600 Jetprinter, Mycronic AB, Täby, Sweden) developed specifically for high-viscosity functional fluids. The technology utilizes a piezo-induced volumetric displacement that forces fluid out through a single 100 μm-wide nozzle at jetting frequencies of up to 300 Hz. The fluid in this case was a SnAgCu 305 solder paste (96.5% Sn, 3% Ag, and 0.5% Cu; from Senju Metal, Senju Hashido-cho 23, Adachi-ku, Tokyo 120-8555, Japan). The paste consists of spherical metallic alloy granules together with an organic resin-based fluid binder in an 82–18 wt% mixture. Droplets with a goal diameter of 227 μm and approximate volume of 2 nL were deposited on the previously deposited Au UBM layers. Positioning accuracy of the deposits is 12 μm (standard deviation). To form a ball connection, the solder paste was reflowed in a commercial vapor phase oven (ASSCON VP800, ASSCON Systemtechnik Elektronik GmbH, Messerschmittring 35, D-86343 Königsbrunn, Germany) utilizing a maximum vapor temperature of 230 °C with a temperature gradient of 0.7 °C s^−1^ between 50 and 100 °C, 1.8 °C s^−1^ between 100–200 °C and 0.8 °C s^−1^ from 200 °C to the peak temperature.

## Results and discussion

### Dielectric filling and Ag UBM pads by inkjet printing

**Figure 4:**
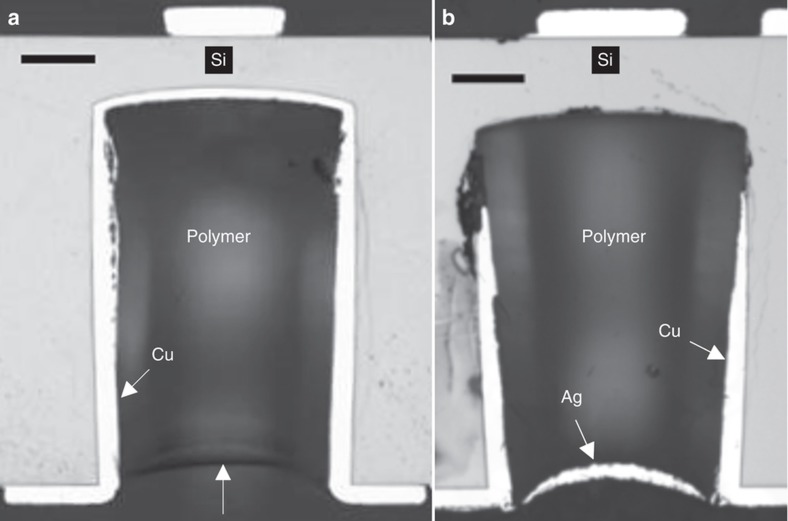
(**a**) Cross-sectional image of the TSV after successful dielectric filling by inkjet printing, where the white arrow at the bottom shows the surface of the dielectric layer. (**b**) Cross-sectional image of the TSV with the Ag UBM layer on top of the dielectric material. Scale bars are 50 μm. TSV, through silicon via; UBM, under bump metallization.

Light microscope images of the hollow copper TSVs that were filled by inkjet printing, after the final baking, showed a sufficient filling level without any voids but with a small concave profile at the surface, as depicted in Figure 4a. The UBM printing results using inkjet technology, as presented in Figure 4b, showed that the silver ink tends to move toward the center of the TSV, resulting in areas on the via edges that are not completely covered with ink and thus may result in reduced electrical connectivity to the plated copper. The minor difference in via depth that is visible in Figures 4a and b is attributed to etch rate variations within a wafer batch during TSV fabrication. The profile of the TSVs appears different in Figures 4a and b; this difference is an artifact resulting from slight tilting and off-center placement of the TSV cross sections. The peeling of the metallization at the bottom of the via in Figure 4b is caused by the preparation of the cross section, that is, by the grinding step. Furthermore, the reason that the connection to the FS RDL is not visible in Figure 4 is that the cross sections are not exactly at the center of the vias because of the limitations in the manual preparation of the cross sections.

### Dielectric filling and Au UBM pad by SIJ printing

**Figure 5:**
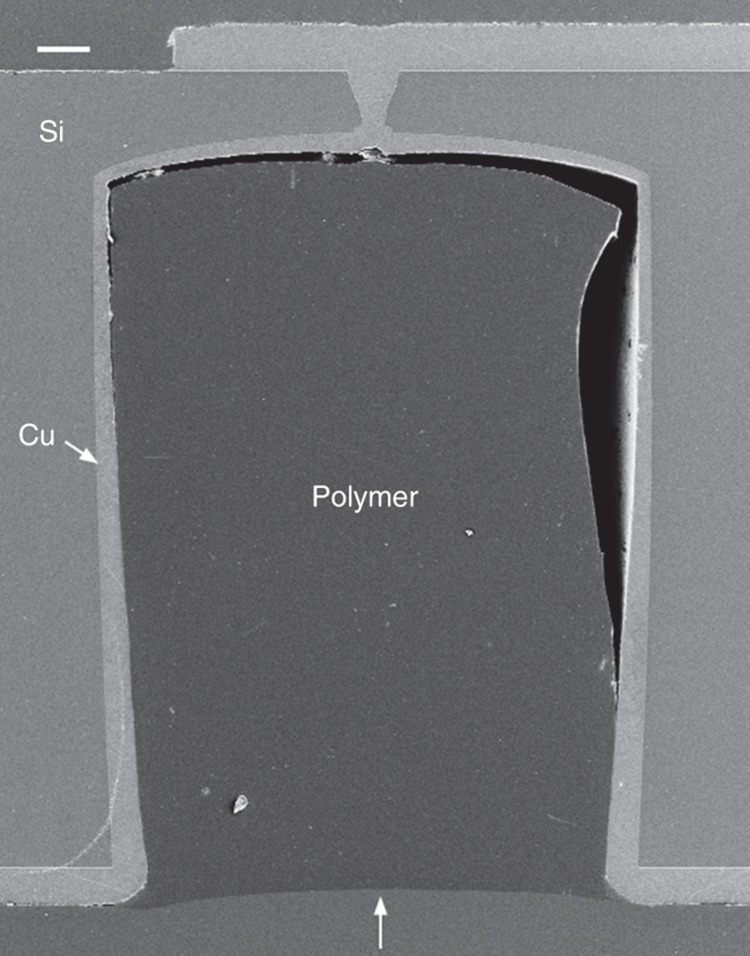
SEM image of a via hole filled using the dispensing mode of super inkjet (SIJ). Scale bar is 20 μm. SEM, scanning electron microscope.

As mentioned in the previous section, SIJ printing was evaluated as an alternative for filling of the via holes. In Figure 5, an SEM micrograph of the cross section of a single via filled by the dispensing mode of SIJ printing is shown. It was concluded that this method can be even faster than using inkjet printing to fill the via holes in applications in which a small number of vias must be filled. In these cases, the filling level of the via hole and the planarity of the dielectric polymer at the via hole surface was improved compared to via holes that were filled by inkjet printing. Furthermore, no voids or other anomalies were observed. However, at the bottom and on one side wall of the via holes, detachment of the dielectric polymer was observed. This could be caused by adhesion problems caused by impurities and moisture as a result of insufficient substrate cleaning (flushing with isopropanol and drying on a hotplate). Poor adhesion could be improved and optimized by adhesion promoters.

**Figure 6:**
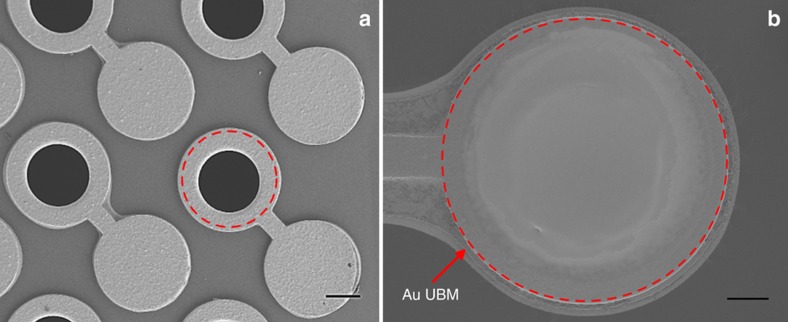
SEM image of (**a**) the top view of the interposer with empty vias and the adjacent pads. (**b**) Via filled with the dielectric and Au UBM dispensed on top by SIJ. Scales are 100 and 50 μm, respectively. SEM, scanning electron microscope; SIJ, super inkjet; UBM, under bump metallization.

By printing circular Au UBM pads using SIJ, complete coverage of the top surface of the filled vias was obtained with a proper connection to the Cu collar around the edge of the vias. In Figure 6a, a dashed circle representing the SEM image of a via with no dielectric filling is shown, and Figure 6b depicts a via filled with the dielectric and Au UBM dispensed on top by SIJ printing, as indicated by the dashed circle.

### Temperature cycle stress test

Electrical testing before and after the temperature cycling (250, 500, 750, and 1000 cycles) was performed using a two-probe DC measurement set-up. No open circuits or significant resistance changes were observed.

**Figure 7:**
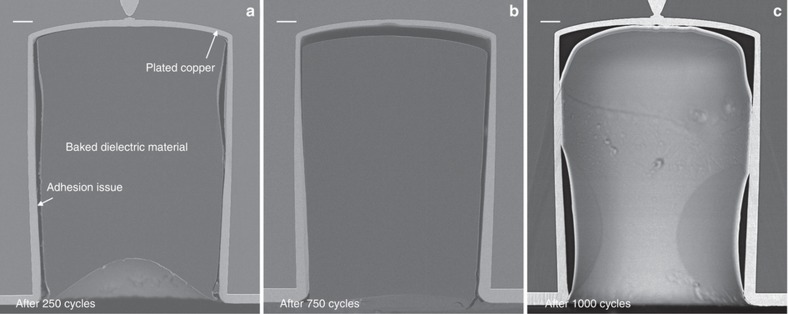
BIB prepared cross-section SEM images of the filled vias after temperature cycling tests with (**a**) 250 cycles, (**b**) 750 cycles, and (**c**) 1000 cycles. Scale bars are 20 μm. In (**b**), the connection between the via metallization and the FS RDL on the top wafer surface cannot be seen because the cross section is not exactly passing through the center of the via. BIB, broad ion beam; FS RDL, frontside redistribution layer; SEM, scanning electron microscope.

Figure 7 illustrates cross sections of the vias filled by SIJ printing with the dielectric polymer after temperature cycling tests with (a) 250 cycles, (b) 750 cycles, and (c) 1000 cycles. Shrinkage and detachment of the dielectric polymer filling was observed; the shrinkage and detachment increased with an increasing number of temperature cycles. In addition, small micro-cracks in the polymer can be seen on the bottom-right corner of the vias in Figures 7a and b. These defects could be due to the adhesion problems, polymer shrinkage, or the mismatch between the CTEs of the polymer and the substrate material. No anomalies in the Si–Cu interfaces were observed.

**Figure 8:**
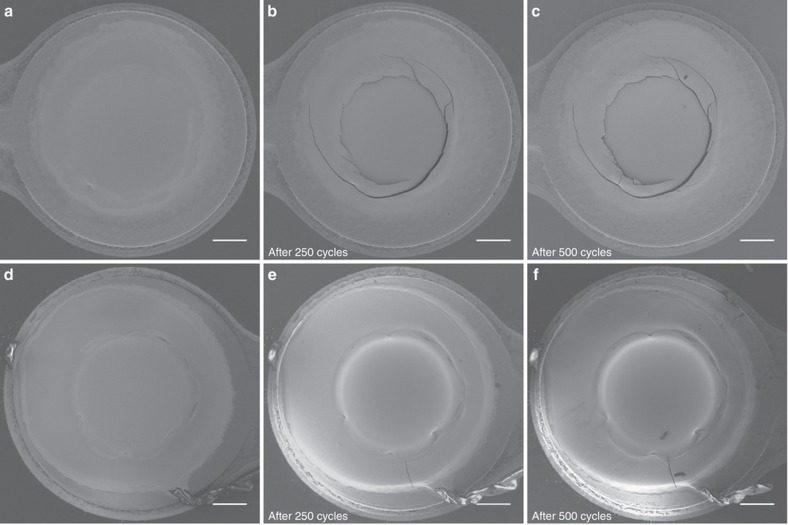
Top view SEM images of the SIJ printed Au UBM pads. Sample 1: (**a**) before temperature cycling and after temperature cycling with (**b**) 250 cycles and (**c**) 500 cycles. Sample 2: (**d**) before temperature cycling and after temperature cycling with (**e**) 250 cycles and (**f**) 500 cycles. Scale bars are 50 μm. SEM, scanning electron microscope; SIJ, super inkjet; UBM, under bump metallization.

Figure 8 shows SEM images of two different samples with Au UBM pads before and after temperature cycling. In Sample 1, after 250 temperature cycles, micro-cracks were observed in a circular pattern at the interface between the Cu and the dielectric polymer (Figure 8b). This failure is likely the same type of micro-crack as that observed in Figures 7a and b. Adding another 250 temperature cycles did not cause any additional defects (Figure 8c). The same observation applies to Sample 2 (Figure 8d), where one micro-crack with the size of ~50 μm was visible after the first 250 cycles (Figure 8e), and no additional visible defects were observed after adding another 250 temperature cycles (Figure 8f).

### Solder ball attachment

**Figure 9:**
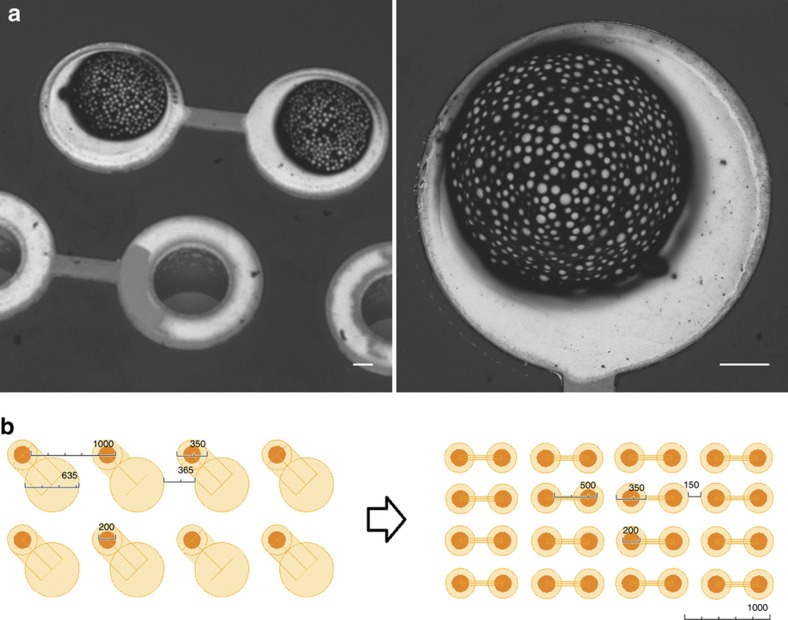
(**a**) SEM images of solder paste deposited on the vias filled with dielectric polymer and covered by printed UBM. Scale bars are 50 μm. (**b**) Schematic of the UBM and solder ball layout on the interposer for demonstrating the increase in I/O density. I/O, input/output; SEM, scanning electron microscope; UBM, under bump metallization.

The solder ball attachment experiments showed successful jetting of the SnAgCu paste on the Au UBM pads, as depicted in Figure 9a. The volume distribution over a series of ball grid arrays using the MY600 Jetprinter depends on the target ball diameter, which is ~5–10% (1 sigma). The stated positioning accuracy is a reliable measure and is sufficient for PCB features with pad sizes as small as 200 μm and pitches as small as under 350 μm. Figure 9b illustrates the dramatic increase in I/O density enabled by our approach, without the need for using next-generation TSV with reduced diameters. The conventional approach^10^ (Figures 2g–i and 9b (left)) allows for a pitch of the solder balls of 1000 μm, resulting in 1 I/Os per mm^2^. The novel approach presented in this work (Figures 2j–l and 9b (right)) enables placing four solder balls per mm^2^ (I/Os per mm^2^), resulting in four times higher I/O density while retaining identical TSV dimensions.

## Conclusion

In this work, inkjet printing and SIJ technology were demonstrated to be available for use in performing the process of dielectric filling of hollow TSVs with an UV-curable hybrid polymer that has a low Young’s modulus. These inkjet methods have been optimized to completely fill the via holes without any voids. The filling process with SIJ produced almost planar surfaces, whereas inkjet-filled vias suffered from a slight concave profile. However, after baking, SIJ-filled vias suffered from partial detachment of the polymer from the via walls, which could be attributed to weak adhesion. It was concluded that the SIJ technology is a better alternative compared to inkjet printing regarding dispensing of metal inks for the UBM pads on top of the vias filled with a dielectric polymer.

Electrical testing before and after temperature cycling experiments showed that temperature cycles of up to 1000 cycles did not cause any open circuits or significantly affect the electrical resistance of the via chains with printed dielectric polymer filling, confirming the findings in Ref. 10. Thus, the difference of the CTEs of Si, Cu, and the dielectric polymer do not appear to affect the electrical functionality of the TSVs. By studying cross sections of the filled vias after temperature cycling, shrinkage and adhesion problems between the dielectric polymer and the bottom and the side walls of the via holes were observed, causing partial detachment of the dielectric polymer in these areas. This effect increased with increasing numbers of temperature cycles (up to 1000 cycles). Dielectric polymers with lower volume shrinkage and a CTE that is better matched to the CTE of the substrate material may be introduced to reduce the observed detachment of the polymer from the sidewalls of the via holes. It was also demonstrated that solder balls can be printed successfully on the interposers, thereby demonstrating the potential for a four-times increased I/O density compared to the same TSV platform used in Ref. 10 but without filling of the hollow vias with the dielectric polymer.

## Figures and Tables

**Table 1:** Material specifications for the polymer dielectric InkOrmo


Material specifications	
Liquid polymer pre-cursor before curing	
Viscosity (mPa s)	12
Hybrid polymer after photocuring (*λ*=365 nm) and hardbake (140–160 °C)	
Volume shrinkage during UV curing (%)	5–7
Volume shrinkage during hardbake (%)	1–2
CTE (20*–*100 °C) (p.p.m. K^−1^)	60
Young’s modulus (GPa)	~1
Hardness (by indentation) (MPa)	~68

**Table 2:** Temperature cycling standards and specifications

Standard conditions	Specifications
JEDEC spec	JESD22-A104
Condition G	−40 °C to +125 °C
Temperature cycle	2 times per hour
Duration	1000 cycles
Soak time	13–14 min

## References

[bib1] Ogutu P, Fey E, Dimitrov N. Superconformal filling of high aspect ratio through glass vias (TGV) for interposer applications using TNBT and NTBC additives. Journal of the Electrochemical Society 2015; 162: D457–D464.

[bib2] Wang Z. 3-D integration and through-silicon vias in MEMS and microsensors. Journal of Microelectromechanical Systems 2015; 24: 1211–1244.

[bib3] Ebefors T, Perttu D. CTE matched interposer and method of making. US Patent No. 9224681. 2015. Available at https://www.google.com/patents/US9224681.

[bib4] Fischer AC, Forsberg F, Lapisa M et al. Integrating MEMS and ICs. Microsystems & Nanoengineering 2015; 1: 15005.

[bib5] Malta D. TSV formation overview. In: Garrou P, Koyanagi M, Ramm P (eds). Handbook of 3D Integration. Wiley-VCH Verlag GmbH & Co. KGaA: Weinheim, Germany, 2014: 65–78.

[bib6] Kälvesten E, Ebefors T, Svedin N et al. Electrical connections in substrates. US Patent No. 7560802. 2009. Available at https://www.google.com/patents/US7560802.

[bib7] Asiatici M, Laakso MJ, Fischer AC et al. Niklaus F. Through silicon vias with invar metal conductor for high-temperature applications. Journal of Microelectromechanical Systems 2017; 26: 158–168.

[bib8] Frank T, Chappaz C, Leduc P et al. Reliability approach of high density through silicon via (TSV). 12th Electronics Packaging Technology Conference; 8–10 Dec 2010; Singapore; 2010: 321–324.

[bib9] Ebefors T, Fredlund J, Jung E et al. Recent results using met-via TSV interposer technology as TMV element in wafer-level through mold via packaging of CMOS biosensors. International Wafer-Level Packaging Conference (IWLPC); 6–7 Nov 2013; San Jose, CA, USA; 2013: 1–8.

[bib10] Yazdani F. Design and direct assembly of 2.5D/3D rigid silicon interposer on PCB. International Symposium on Microelectronics 13–16 Oct 2014; San Diego, CA, USA; 2014: 783–786.

[bib11] Ebefors T, Fredlund J, Perttu D et al. The development and evaluation of RF TSV for 3D IPD applications. 2013 IEEE International 3D Systems Integration Conference (3DIC); 2–4 Oct 2013; San Francisco, CA, USA; 2013: 1–8. doi: 10.1109/3DIC.2013.6702382.

[bib12] Rathjen A, Bergmann Y, Krüger K. Feasibility study: Inkjet filling of through silicon vias (TSV). NIP & Digital Fabrication Conference; 9–13 Sep 2012; Quebec City, Canada; 2012: 456–460.

[bib13] Khorramdel B, Mäntysalo M. Fabrication and electrical characterization of partially metallized vias fabricated by inkjet. Journal of Micromechanics and Microengineering 2016; 26: 45017.

[bib14] Khorramdel B, Laurila MM, Mantysalo M. Metallization of high density TSVs using super inkjet technology. IEEE 65th Electronic Components and Technology Conference (ECTC); 26–29 May 2015; San Diego, CA, USA; 2015: 41–45.

[bib15] Khorramdel B, Mantysalo M. Inkjet filling of TSVs with silver nanoparticle ink. Proceedings of the 5th Electronics System-Integration Technology Conference (ESTC); 16–18 Sep 2014; Helsinki, Finland; 2014: 1–5.

[bib16] Eiroma K, Viljanen H. Application of inkjet printing for 3D integration. NIP & Digital Fabrication Conference; 28 Sep–1 Oct 2015; Protland, OR, USA; 2015: 195–200.

[bib17] Quack N, Sadie J, Subramanian V et al. Through silicon vias and thermocompression bonding using inkjet-printed gold nanoparticles for heterogeneous MEMS integration. Transducers & Eurosensors XXVII: The 17th International Conference on Solid-State Sensors, Actuators and Microsystems (TRANSDUCERS & EUROSENSORS XXVII); 16–20 Jun 2013; Barcelona, Spain; 2013: 834–837.

[bib18] Sadie J, Quack N, Wu MC et al. Droplet-on-demand Inkjet-filled through-silicon Vias (TSVs) as a pathway to cost-efficient chip stacking. 46th International Symposium on Microelctronics; 30 Sep–3 Oct 2013; Orlando, FL, USA; 2013: 866–871.

[bib19] Bouchoucha M, Chausse P, Moreau S et al. Reliability study of 3D-WLP through silicon via with innovative polymer filling integration. IEEE 61st Electronic Components and Technology Conference (ECTC); 31 May–3 Jun 2011; Lake Buena Vista, FL, USA; 2011: 567–572.

[bib20] Bouchoucha M, Chapelon L-L, Chausse P et al. Through silicon via polymer filling for 3D-WLP applications. 3rd Electronics System-Integration Technology Conference (ESTC); 13–16 Sep 2010; Berlin, Germany; 2010: 1–4; doi:10.1109/ESTC.2010.5642998.

[bib21] Chausse P, Bouchoucha M, Henry D et al. Polymer filling of medium density through silicon via for 3D-packaging. 11th Electronics Packaging Technology Conference; 9–11 Sep 2009; Singapore; 2009: 790–794.

[bib22] Soltani A, Khorramdel Vahed B, Mardoukhi A et al. Laser sintering of copper nanoparticles on top of silicon substrates. Nanotechnology 2015; 27: 35203. 10.1088/0957-4484/27/3/035203 26650565

[bib23] Niittynen J, Sowade E, Kang H et al. Comparison of laser and intense pulsed light sintering (IPL) for inkjet-printed copper nanoparticle layers. Scientific Reports 2015; 5: 8832. 25743631 10.1038/srep08832 PMC4351538

[bib24] Murata K, Masuda K. Super inkjet printer technology and its properties. Convertech e-Print 2011; 1: 108–111.

