# Supplementary material for: Inkjet printing technology for increasing the I/O density of 3D TSV interposers
Source: Microsyst Nanoeng. 2017 Apr 10;3:17002. doi: 10.1038/micronano.2017.2 (PMC6445024; doi:10.1038/micronano.2017.2)
Supplement: Supplementary Tables [file micronano20172-s1.pdf]

Supplementary file

Inkjet printing technology for increasing the I/O density of 3D TSV interposers

Behnam Khorramdel[1], Jessica Liljeholm[2,3], Mika-Matti Laurila[1], Toni Lammi[1], Gustaf Mårtensson[4,5], Thorbjörn Ebefors[2], Frank Niklaus[3] and Matti Mäntysalo[1]

Microsystems & Nanoengineering (2017) **3,** 17002; doi:10.1038/micronano.2017.2; Published online: 10 April 2017

Table S1 Printing parameters for printing fresh InkOrmo polymer with the Dimatix

Cartridge setting	
Jetting voltage (Volts)	27.3
Cartridge temperature (in C)	30
Substrate temperature (in C)	28
Meniscus vacuum (inches H_2O)	4.0
Cartridge print height (mm)	0.8

Table S2 Printing parameters used for printing the UBM pads with SIJ

Waveform	Sine wave
Amplitude [V]	453
Bias (DC) [V]	0 (on top of dielectric)
ON speed [$mm\,s^{-1}$]	0.30
Frequency [Hz]	70
Spiral pitch [μm]	15
Number of layers	~60

[1]Department of Electronics and Communications Engineering, Tampere University of Technology, Korkeakoulunkatu 3, Tampere 33720, Finland; [2]Silex Microsystems AB, Bruttovägen 1, Järfälla/Stockholm SE-175 26, Sweden; [3]KTH Royal Institute of Technology, Micro and Nanosystem, Osquldas väg 10, 10444 Stockholm, Sweden; [4]EMSL, Chalmers University of Technology, Kemivägen 9, 41296 Göteborg, Sweden and [5]Mycronic AB, Nytorpsvägen 9, 18303 Täby, Sweden
Correspondence: Behnam Khorramdel (behnam.khorramdel@tut.fi) or Frank Niklaus (frank.niklaus@ee.kth.se) or Matti Mäntysalo (matti.mantysalo@tut.fi)
